# The ethics of animal research: a survey of pediatric health care workers

**DOI:** 10.1186/s13010-014-0020-7

**Published:** 2014-12-30

**Authors:** Ari R Joffe, Meredith Bara, Natalie Anton, Nathan Nobis

**Affiliations:** Department of Pediatrics, University of Alberta, 8440 112 Street, Edmonton, Alberta T6G 2B7 Canada; John Dossetor Health Ethics Center, University of Alberta, 5-16 University Terrace, Edmonton, Alberta T6G 2T4 Canada; Faculty of Medicine and Dentistry, University of Alberta, 8440 112 Street, Edmonton, Alberta T6G 2B7 Canada; Department of Philosophy, Morehouse College, Atlanta, GA USA; Community Health & Preventive Medicine, Morehouse School of Medicine, Atlanta, GA USA; 4-546 Edmonton Clinic Health Academy, 11405 87 Ave, Edmonton, Alberta Canada

**Keywords:** Survey, Animals, Animal research, Ethics

## Abstract

**Introduction:**

Pediatric health care workers (HCW) often perform, promote, and advocate use of public funds for animal research (AR). We aim to determine whether HCW consider common arguments (and counterarguments) in support (or not) of AR convincing.

**Design:**

After development and validation, an e-mail survey was sent to all pediatricians and pediatric intensive care unit nurses and respiratory therapists (RTs) affiliated with a Canadian University. We presented questions about demographics, support for AR, and common arguments (with their counterarguments) to justify the moral permissibility (or not) of AR. Responses are reported using standard tabulations. Responses of pediatricians and nurses/RTs were compared using Chi-square, with *P <* .05 considered significant.

**Results:**

Response rate was 53/115(46%) (pediatricians), and 73/120(61%) (nurses/RTs). Pediatricians and nurses/RTs are supportive of AR. Most considered ‘benefits arguments’ sufficient to justify AR; however, most acknowledged that counterarguments suggesting alternative research methods may be available, or that it is unclear why the same ‘benefits arguments’ do not apply to using humans in research, significantly weakened ‘benefits arguments’. Almost all were not convinced of the moral permissibility of AR by ‘characteristics of non-human-animals arguments’, including that non-human-animals may not be sentient, or are simply property. Most were not convinced of the moral permissibility of AR by ‘human exceptionalism’ arguments, including that humans have more advanced mental abilities, are of a special ‘kind’, can enter into social contracts, or face a ‘lifeboat situation’. Counterarguments explained much of this, including that not all humans have these more advanced abilities [the argument from species overlap], and that the notion of ‘kind’ is arbitrary [e.g., why are we not of the kind ‘sentient animal’ or ‘subject-of-a-life’]. Pediatrician and nurse/RT responses were similar.

**Conclusions:**

Most respondents were not convinced of the moral permissibility of AR when given common arguments and counterarguments from the literature. HCW should seriously consider arguments on both sides of the AR debate.

## Introduction

How we justify animal research (AR) is a controversial issue. Nevertheless, this is an important issue in pediatrics for several reasons. Pediatric health care workers (HCW) often perform (and are expected to perform) AR, promote AR directly with trainees and indirectly as role models, and advocate for use of public funds (from granting agencies and charitable foundations) toward pediatric related AR. Therefore, it might be expected that the arguments for and against AR are well settled. However, standard ethical arguments and counterarguments for AR are rarely discussed in the medical literature, and it is likely that many are not aware of the debate.

It is necessary to clarify our definitions prior to discussing the AR debate here. First, AR refers to research that is harmful [i.e. detrimental to some interest the being has, such as the interest in maintaining life and bodily integrity, and avoiding pain and frustration], non-therapeutic [does not aim at restoring the health of a research subject with *prior* injury/disease], and non-consensual [conducted with subjects who have not voluntarily agreed to participate] [1]. In other words, the procedures would be judged unethical if done with any non-consenting human subjects or done in a non-research setting [1]. Second, ‘animal’ in AR refers to those animals that are sentient, that is, capable of experiencing suffering. This is generally agreed to include at least mammals and birds [2,3]. Third, AR is a moral issue because animals are harmed in experimentation, from such things as confinement [with boredom, loneliness, and frustration], fear [from handling], pain [from blood collection, and diseases induced], and early death [4,5]. The question debated is this: is any or all AR that involves seriously harming animals morally permissible?

There are several general types of arguments used to justify AR [5-7]. First are what we call “benefits arguments”: AR benefits humans greatly, or AR is necessary for human benefit, or there are no alternatives to AR for human benefit. These are the most common justifications given by pro-AR groups [8-10]. Second are what we call “characteristics of non-human-animals (NHAs) arguments”: animals harm other animals, or are not sentient, or are property. These are arguments that led to the initial development of AR and its legal regulation [11]. Third are what we call “human exceptionalism arguments”: compared to NHAs, humans have more advanced abilities, or are of a special kind, or can enter into contracts; or humans must sacrifice NHAs in their lifeboat to save other humans [12-15]. Interestingly, the first two types actually rely on the third type of argument; for example, to justify using animals [as necessary] for human benefits or as property requires an argument for why humans cannot be used in the same way [12]. All these arguments have replies that have been discussed in the philosophical literature. However, the debate about the cogency of these arguments and counterarguments is rarely discussed in the medical literature. We aimed to determine not simply whether pediatric HCWs support AR, but whether they thought the usual arguments (and counterarguments) in support (or not) of AR were convincing.

## Methods

### Questionnaire administration

All pediatricians and pediatric intensive care unit nurses and respiratory therapists (RTs) who are affiliated with the Canadian University were e-mailed the survey using an electronic, secure, survey distribution and collection system (REDCap, Research Electronic Data Capture). A cover letter stated that “we very much value your opinion on this important issue” and that the survey was anonymous and voluntary. We also offered the incentive that if the response rate was at least 70% we would donate $1000 to the Against Malaria Foundation or the PICU Social Committee. Non-responders were sent the survey by e-mail at 3-week intervals for up to 3 additional mailings.

### Questionnaire development

The development of the questionnaire followed published recommendations [16]. To generate items for the questionnaire, we searched Medline from 1980 to 2012 for articles about the ethics of AR. We also searched the Journal of Applied Philosophy, and reviewed recent textbooks on the topic of the ethics of AR. This was followed by collaborative creation of the background section and questions for the survey by the authors. Content and construct validation were done using a table of specifications filled out by experts including two ethics philosophy professors, and two pediatricians. Face and content validation were done by pilot testing of the survey by non-medical, university-educated lay people (n = 9), pediatricians (n = 2), pediatric intensive care nurses (n = 2), and an ethics professor (n = 1). Each pilot test was followed by an informal semi-structured interview by one of the authors to ensure clarity, realism, validity, and ease of completion of the questionnaire. A published clinical sensibility tool was also used for the expert and pilot testing [16]. After minor modifications, the survey was approved by all authors.

### Questionnaire content

The background section described the situation: “In this survey, ‘animals’ means: mammals, such as mice, rats, dogs, and cats. It has been estimated that over 100 million animals are used in the world for research each year. There are many good reasons to justify AR, which is the topic of this survey. Nevertheless, some people argue that these animals are harmed in experimentation, because their welfare is worsened. In this survey, ‘harmful’ means such things as: pain, suffering (disease/injury, boredom, fear, confinement), and early death. We value your opinion on the very important issue of the ethical dimension of AR”.

We presented demographic questions, 3 questions about support for AR, and 12 arguments with their counterarguments to consider. The arguments and counterarguments were introduced as follows: “a) First, we give you an argument to justify harmful AR, and we ask you if you agree with that argument; b) then we give some responses to the argument, and we ask if you think each response would make it harder for someone to justify harmful AR using the initial argument (i.e. would make the initial argument much less convincing). All the arguments and responses in this survey are those commonly made in the literature on AR”. When each argument was presented it was followed with the question: “Is this a good enough reason to justify using animals in medical research?” When the responses to an argument were presented, they were introduced with the question: “Do any of the following responses make it harder for someone to justify AR using Argument X [i.e. make Argument X much less convincing]?” Responses were in a ‘yes’ or ‘no’ format.

### Ethics approval

The study was approved by the health research ethics board of our university, and return of a survey was considered consent to participate.

### Statistics

We used REDCap as our survey management tool [17]. This web-based tool allows anonymous survey responses to be collected, and later downloaded into an SPSS database for analysis. Responses were analyzed by using standard tabulations. Variables expressed as percentages were used to report the proportion of respondents with different answers. The responses of the two predefined groups, pediatricians and PICU nurses/RTs, were compared using the Chi-square statistic, with *P <* .05 after Bonferroni correction for multiple comparisons considered significant.

## Results

### Pediatrician responses

#### Demographics

The response rate was 53/115 (46%). Demographics included: males 24/51 (47%); age <35 yr 5/52 (10%), 35-44 yr 20/52 (39%), and >44 yr 27/52 (52%); those who have done AR in the past 19/52 (37%), currently do AR 3/52 (6%), and never done AR 31/52 (58%).

#### Benefits arguments and counterarguments

Responses are shown in Table 1. A majority accepted the benefits arguments, except for the “humans naturally need to seek knowledge” argument. However, many found the counterarguments convincing, including those who initially responded that the argument was enough to justify AR. Most were convinced by counterarguments with suggestions about the availability of alternative research methods that do not use animals, including statements that more effort should be devoted to developing alternative research methods. A significant minority also thought counterarguments convincing that state that “if great human benefits justify using animals in medical research, this should also justify using humans in the same medical research”.

**Table 1:** **Responses to the ‘benefits’ arguments and counterarguments**

**Respondent group**	**Is this a good enough reason to justify using animals in medical research?**		**Do any of the following responses make it harder for someone to justify animal research using the argument [i.e. make the argument much less convincing]?**		**Of those convinced by the argument: proportion who judged the counterargument to make the argument much less convincing**
	**Yes**	**No**	**Yes**	**No**	
**Argument (A)/counterargument (CA)**					
A1. Animal experimentation benefits humans greatly.					
Pediatrician	35/51 (69%)	16/51 (31%)			
Nurse/RT	35/73 (48%)	38/73 (52%)			
CA: If great human benefits justify using animals in medical research, this should also justify using humans in the same medical research.					
Pediatrician			19/51 (37%)	32/51 (63%)	8/35 (23%)
Nurse/RT			37/71 (52%)	34/71 (48%)	15/34 (44%)
CA: If animals can experience pain and suffering, it remains unclear why we morally may use them in experiments for human benefit.					
Pediatrician			31/51 (61%)	20/51 (39%)	17/35 (49%)
Nurse/RT			51/71 (72%)	20/71 (28%)	20/34 (59%)
A2: Animal experimentation is necessary for human benefit.					
Pediatrician	29/51 (57%)	22/51 (43%)			
Nurse/RT	31/69 (45%)	38/69 (55%)			
CA: More humans would benefit if the money spent on animal experiments was instead devoted to humanitarian aid (for example, in developing countries).					
Pediatrician			24/50 (48%)	26/50 (52%)	10/26 (38%)
Nurse/RT			33/69 (48%)	36/69 (52%)	10/30 (33%)
CA: There are now alternative experimental methods that do not use animals and that allow science to advance.					
Pediatrician			40/49 (82%)	9/49 (18%)	21/26 (81%)
Nurse/RT			62/68 (91%)	6/68 (9%)	26/30 (87%)
CA: It is unclear why the statement animal experimentation is necessary for human benefits justifies animal experiments, but the statement human experimentation is necessary for human benefits does not justify the same experiments on humans.					
Pediatrician			24/49 (49%)	25/49 (51%)	5/26 (19%)
Nurse/RT			46/67 (69%)	21/67 (31%)	18/30 (60%)
A3: There are no alternatives to animal experimentation.					
Pediatrician	24/48 (50%)	24/48 (50%)			
Nurse/RT	30/67 (45%)	37/67 (55%)			
CA: Researchers have not looked hard enough for alternatives to animal experimentation. For example, since using animals to test drugs has been required by law, researchers may have assumed that there is no other way.					
Pediatrician			34/48 (71%)	14/48 (29%)	14/24 (58%)
Nurse/RT			50/65 (77%)	15/65 (23%)	21/28 (75%)
CA: If more effort was devoted to developing alternative research methods that do not use animals, animal experimentation may not be necessary anymore.					
Pediatrician			36/48 (75%)	12/48 (25%)	14/24 (58%)
Nurse/RT			56/65 (86%)	9/65 (14%)	23/28 (82%)
A4: Humans naturally need to seek knowledge.					
Pediatrician	2/46 (4%)	44/46 (96%)			
Nurse/RT	10/62 (16%)	52/62 (84%)			
CA: This can justify almost anything, including harmful experiments on humans against their will, in order to gain knowledge.					
Pediatrician			35/47 (75%)	12/47 (26%)	1/2 (50%)
Nurse/RT			44/62 (71%)	18/62 (29%)	5/10 (50%)
CA: We have learned a great deal from earthquakes, fires and warfare; but, this does not justify recreating these things in order to gain more knowledge.					
Pediatrician			33/47 (70%)	14/47 (30%)	1/2 (50%)
Nurse/RT			48/63 (76%)	15/63 (24%)	7/10 (70%)

#### Characteristics of NHAs arguments and counterarguments

Responses are shown in Table 2. Almost all respondents were not convinced by these arguments. For most, the stated counterarguments explained their lack of acceptance of the initial arguments.

**Table 2:** **Responses to the ‘characteristics of non-human-animals’ arguments and counterarguments**

**Respondent group**	**Is this a good enough reason to justify using animals in medical research?**		**Do any of the following responses make it harder for someone to justify animal research using the argument [i.e. make the argument much less convincing]?**		**Of those convinced by the argument: proportion who judged the counterargument to make the argument much less convincing**
	**Yes**	**No**	**Yes**	**No**	
**Argument (A)/counterargument (CA)**					
A1. Animals harm other animals.					
Pediatrician	1/47 (2%)	46/47 (98%)			
Nurse/RT	4/63 (6%)	59/63 (94%)			
CA: It is unclear why we should take this (we may harm animals) as moral advice from animals, but not take other moral advice from animals (for example, animals rape and kill members of their own species would mean we may rape and kill humans). In other words, animals are not qualified to give moral advice.					
Pediatrician			31/47 (66%)	16/47 (34%)	1/1 (100%)
Nurse/RT			37/61 (61%)	24/61 (39%)	2/4 (50%)
A2: Animals cannot really feel anything. They are simply living machines.					
Pediatrician	0/45 (0%)	45/45 (100%)			
Nurse/RT	1/63 (2%)	62/63 (98%)			
CA: This would mean that a pet cat or dog is simply a living machine, without any feelings like happiness, sadness, fear or pain.					
Pediatrician			33/46 (72%)	13/46 (28%)	-
Nurse/RT			36/62 (58%)	26/62 (42%)	0/1 (0%)
A3: Animals are property					
Pediatrician	1/42 (2%)	41/42 (98%)			
Nurse/RT	2/58 (3%)	56/58 (97%)			
CA: Since animals can desire things, intentionally act to fulfill those desires, and can understand (even dimly) that it is me that wants something and is trying to get it, they are not simply property.					
Pediatrician			30/41 (73%)	11/41 (27%)	0/1 (0%)
Nurse/RT			36/59 (61%)	23/59 (39%)	0/2 (0%)

#### Human exceptionalism arguments and counterarguments

Responses are shown in Table 3. A large majority of respondents did not accept these arguments as good enough to justify AR. For most, the stated counterarguments explained their lack of acceptance of the initial arguments. The arguments, that humans have more advanced abilities, or are of a special kind, were considered good enough reasons to justify AR for only 4 (9%) and 11 (26%) of respondents respectively. The arguments about contractualism and lifeboat-ethics were considered good enough reasons to justify AR for only 2 (5%) and 9 (21%) of respondents respectively.

**Table 3:** **Responses to the ‘human exceptionalism’ arguments and counterarguments**

**Respondent group**	**Is this a good enough reason to justify using animals in medical research?**		**Do any of the following responses make it harder for someone to justify animal research using the argument [i.e. make the argument much less convincing]?**		**Of those convinced by the argument: proportion who judged the counterargument to make the argument much less convincing**
	**Yes**	**No**	**Yes**	**No**	
**Argument (A)/counterargument (CA)**					
A1.Humans have more advanced mental abilities than animals, like knowing right from wrong, having empathy, planning for the future, and being able to read and talk.					
Pediatrician	4/45 (9%)	41/45 (91%)			
Nurse/RT	9/61 (15%)	52/61 (85%)			
CA: Not all humans have these abilities. Babies, infants, and severely brain damaged children or adults (for example, with very advanced Alzheimers) do not have these abilities. Some animals may have more abilities than these humans.					
Pediatrician			28/44 (64%)	16/44 (36%)	1/4 (25%)
Nurse/RT			39/61 (64%)	22/61 (36%)	3/9 (33%)
CA: This means having superior abilities [humans] justifies actively harming those with inferior abilities [animals]. It is unclear why, if animals can experience pain and suffering, having lower mental abilities makes it acceptable to use them in experiments. For example, sometimes humans with superior abilities [adults] have many obligations to those with inferior abilities [children].					
Pediatrician			30/44 (68%)	14/44 (32%)	1/4 (25%)
Nurse/RT			35/61 (57%)	26/61 (43%)	2/9 (22%)
A2: Humans are a special kind or group. We care more about this kind, and have more obligations to this kind.					
Pediatrician	11/43 (26%)	32/43 (75%)			
Nurse/RT	9/61 (15%)	52/61 (85%)			
CA: Imagine there is a more advanced species than humans. This would mean that they are justified in using humans in experiments, because they care more about their own kind.					
Pediatrician			28/42 (67%)	14/42 (33%)	4/11 (36%)
Nurse/RT			30/60 (50%)	30/60 (50%)	3/8 (38%)
CA: Maybe humans are of the kind ‘able to experience suffering and pleasure’ (sentient being). If so, our kind includes animals.					
Pediatrician			23/41 (56%)	18/41 (44%)	3/11 (27%)
Nurse/RT			34/61 (56%)	27/61 (44%)	1/8 (13%)
CA: Maybe humans are of the kind ‘able to have experiences, memories, and preferences’ (subject of a life). If so, our kind includes animals.					
Pediatrician			24/42 (57%)	18/42 (43%)	3/11 (27%)
Nurse/RT			35/60 (58%)	25/60 (42%)	2/8 (25%)
CA: It is unclear why caring more about someone justifies harming those we care less about. For example, in the past this argument was used to justify prejudice (for example, slavery) against those we cared less about, who were considered not of our own kind.					
Pediatrician			32/42 (76%)	10/42 (24%)	6/11 (55%)
Nurse/RT			45/60 (75%)	15/60 (25%)	5/8 (63%)
A3: We have moral duties only to those who can agree to the same duties. This is like a contract between people in society. Since animals cannot enter into this contract with humans, we do not have moral duties to animals.					
Pediatrician	2/43 (5%)	41/43 (95%)			
Nurse/RT	5/61 (8%)	56/61 (92%)			
CA: This would mean we have no direct moral duties to humans who cannot enter into this contract. For example, babies, and severely brain-damaged people.					
Pediatrician			31/42 (74%)	11/42 (26%)	0/2 (0%)
Nurse/RT			33/60 (55%)	27/60 (45%)	1/5 (20%)
A4: Evolution, and our nature, dictates that we must make sure we survive as a species.					
Pediatrician	7/42 (17%)	35/42 (83%)			
Nurse/RT	18/60 (30%)	42/60 (70%)			
CA: It is unclear why what we evolved to do [survive at all costs] is what we morally should do. In other words, evolution does not take moral considerations into account.					
Pediatrician			28/41 (68%)	13/41 (32%)	2/7 (29%)
Nurse/RT			29/57 (51%)	28/57 (49%)	5/16 (31%)
CA: Research is unlikely to save our species; it is for the benefit of some humans, not the whole species (which is what evolution is about).					
Pediatrician			23/41 (56%)	18/41 (44%)	1/7 (14%)
Nurse/RT			27/60 (45%)	33/60 (55%)	6/16 (38%)
A5: We must sacrifice one (animals) in order to save another (humans). This is like being in a lifeboat on the ocean where we must throw one overboard or the lifeboat will sink.					
Pediatrician	9/42 (21%)	33/42 (79%)			
Nurse/RT	15/58 (26%)	43/58 (74%)			
CA: Most people would throw a dog overboard to save humans in the lifeboat; but, this does not mean that the dog can be used in experiments. For example, some might throw an elderly man overboard to save their children in the lifeboat; but, this does not mean elderly men can be used for experiments.					
Pediatrician			26/41 (63%)	15/41 (37%)	4/9 (44%)
Nurse/RT			36/59 (61%)	23/59 (39%)	5/15 (33%)

#### General questions

We asked at the beginning and again at the end of the survey if “in order to achieve human benefits, research that results in harm to animals should be supported”. At the beginning, pediatricians responded ‘yes’ in 32/52 (62%); at the end in 24/41 (59%) [p = 0.77]. Finally, we asked “what is it about vulnerable humans (for example, babies, severely brain damaged people, people with very advanced Alzheimer’s) that makes it wrong to use them in experiments?” Pediatricians responded as follows: 5/42 (12%) “these vulnerable humans are able to experience things like pleasure, joy, happiness, sadness, pain, and suffering”, 11/42 (26%) “these humans are vulnerable to physical and psychological harm; using them in experiments is harmful for them”; 5/42 (12%) “we care about them”; and 21/42 (50%) “they are still human”.

### Nurses and respiratory therapists responses

#### Demographics

The response rate was 73/120 (61%). Demographics included: males in 12/72 (17%); nurses in 56/68 (82%) and RTs in 12/68 (18%); age <35 yr in 45/73 (61%), 35-44 yr in 11/73 (15%), and >44 yr in 17/73 (23%); have done AR in the past in 8/73 (11%), currently do AR in 0, and never done AR in 65/73 (89%).

#### Benefits arguments and counterarguments

Responses are shown in Table 1. Almost half of respondents (45-48%) accepted the benefits arguments, except for the “humans naturally need to seek knowledge” argument. However, most found several of the counterarguments convincing, including those who initially responded that the argument was enough to justify AR. Most were convinced by counterarguments with suggestions about the availability of alternative research methods that do not use animals, including statements that more effort should be devoted to developing alternative research methods. A majority (52-69%) also thought counterarguments convincing that state that “if great human benefits justify using animals in medical research, this should also justify using humans in the same medical research”.

#### Characteristics of NHAs arguments and counterarguments

Responses are shown in Table 2. Almost all respondents were not convinced by these arguments. For most, the stated counterarguments explained their lack of acceptance of the initial arguments.

#### Human exceptionalism arguments and counterarguments

Responses are shown in Table 3. A large majority of respondents did not accept any of these arguments as good enough to justify AR. For most, the stated counterarguments explained much of their lack of acceptance of the initial arguments. The arguments that humans have more advanced abilities, or are of a special kind, were each considered good enough reasons to justify AR for only 9 (15%) of respondents. The arguments about contractualism and lifeboat-ethics were considered good enough reasons to justify AR for only 5 (8%) and 15 (26%) of respondents respectively.

#### General questions

We asked at the beginning and again at the end of the survey if “in order to achieve human benefits, research that results in harm to animals should be supported”. At the beginning, nurses/RTs answered ‘yes’ in 31/72 (43%); at the end in 19/59 (32%) [p = 0.20]. Finally, when asked about what makes it wrong to use vulnerable humans in experiments, nurses/RTs responded as follows: 12/59 (20%) “these vulnerable humans are able to experience things like pleasure, joy, happiness, sadness, pain, and suffering”, 20/59 (34%) “these humans are vulnerable to physical and psychological harm; using them in experiments is harmful for them”; 5/59 (9%) “we care about them”; and 22/59 (37%) “they are still human”.

### Comparisons of pediatrician and nurses/RT responses

No statistically significant differences were found between the subgroups in responses to any of the three categories of arguments/counterarguments. At the beginning (p = 0.036) and end (p = 0.009) of the survey, nurses/RTs were less likely to support AR. There were no statistically significant differences in the response to the final question about what makes it wrong to use vulnerable humans in experiments.

## Discussion

There are four main findings from this survey. First, pediatricians (62%) and nurses/RTs (43%) are supportive of AR. Second, ‘benefits arguments’ were usually thought sufficient to justify AR; however, when confronted with counterarguments pointing out that alternative research methods may be available, or suggesting that it is unclear why the same ‘benefits arguments’ do not apply to using humans in research, most were not as convinced. Third, almost all respondents were not convinced by ‘characteristics of NHAs arguments’ , including that NHA may not be sentient, or are simply property. Fourth, a large majority of respondents were not convinced by the main arguments that have been offered in favor of ‘human exceptionalism’ , those arguments that justify AR due to the benefits, but that claim the same benefits do not justify human research. These include arguments that humans have more advanced mental abilities than NHAs, that humans are of a special ‘kind’ , that humans can enter into social contracts, or that we face a lifeboat situation where human interests trump NHA interests [12-15]. Finally, the counterarguments offered explained much of the respondents’ lack of acceptance of ‘human exceptionalism’ , including pointing out that not all humans have these more advanced abilities [the so-called ‘argument from species overlap’ or ‘argument from marginal cases’], [18-20] and that the notion of ‘kind’ is vague [e.g. why are we not of the kind ‘sentient animal’ or ‘subject-of-a-life’] [21] and has been used in the past to justify prejudice against those society cared less about [22]. There are important implications of these findings for public and HCW acceptance of AR (Table 4).

**Table 4:** **Possible important implications of the findings from this survey**

**Respondents’ opinions placed in the context of recent philosophy literature** [6]	**Possible implication(s)**
‘Benefits arguments’ were initially convincing. However, most respondents recognized that there is a missing premise: a reason needs to be given to justify respecting human interests in avoiding suffering differently from NHA similar interests in avoiding suffering [12].	HCWs’ support for AR may not be based on cogent philosophical rationales, and rather may be based on group membership effects, with commitment to a current ‘Kuhnian’ scientific research paradigm of AR [23]
Most were not convinced by ‘human exceptionalism arguments’, despite these being the main pro-AR arguments in the literature [12-15]	
Common counterarguments in the philosophy literature explain this well:	
a) the ‘argument from species overlap’ [sometimes called the ‘argument from marginal cases’], [18-20]	
b) suggestions that NHAs may be ‘subjects-of-a-life’ [i.e. experiencing subjects of their own life] and ‘sentient’ [21,22], and	
c) suggestions that species membership may not be morally relevant [‘speciesism’ arguments that draw a parallel to previous prejudices such as racism and sexism, where like-interests in one group are disregarded compared to another group] [22].	
Almost none thought that NHAs should be considered property, or that NHAs are not sentient. Importantly, legal protections for NHAs are based on the assertion that these NHAs are property [24], and belief that NHAs are sentient is the basis for the counterarguments that question the moral permissibility of AR.	Current AR animal protection practices may not be in line with HCWs’ beliefs about NHAs.
Most respondents were supportive of AR even after considering the arguments and counterarguments given.	Social science research is needed to determine why philosophical argumentation does not translate into practical behavior change [25].
Counterarguments suggesting that “researchers have not looked hard enough for alternatives to animal experimentation” and “if more effort was devoted to developing alternative research methods that do not use animals, animal experimentation may not be necessary anymore” were convincing for most respondents. Thus, some of the support for AR is based on the belief that there are no alternative research methods.	Focus on the return on investment from AR and alternative research methods may help people in considering the ethics of AR [26,27] The translation rates into human benefit (i.e. accuracy of research models) should be determined for both AR and alternative research methods in order to inform the debate about AR.

Previous public surveys have generally asked only whether people support AR for human benefit, and not asked people to evaluate their reasons for supporting (or not) AR [28]. For example, the Eurobarometer asks “scientists should be allowed to experiment on animals like dogs and monkeys if this can help sort out human health problems”; in 2010, 44% of Europeans responded ‘agree’ and 37% ‘disagree’ [29]. In the UK, Ipsos MORI in 2012 determined that most people (85%) are ‘conditional acceptors’ of AR; they accept AR “so long as it is for medical research purposes” or “for life-threatening diseases” or “where there is no alternative”, considering AR as a “necessary evil” for human benefit [30]. Even so, 37% were objectors (including 53% of those age 15-24 yr); they respond that they “do not support the use of animals in any experiments because of the importance I place on animal welfare” or “the government should ban all experiments on animals for any form of research” [30]. In addition, most (76%) agreed that “there needs to be more research into alternatives to animal experimentation” [30]. In the United States in 2011 the Gallup’s Values and Beliefs survey found that when asked whether medical testing on animals is morally acceptable or morally wrong, 43% (including 54% of those age 18-29 yr) responded ‘morally wrong’ [31]. Two recent surveys did ask for some elaboration on reasons for supporting AR. In a survey in Sweden including rheumatoid arthritis patients and scientific expert members of research ethics boards, most respondents agreed to AR for at least some type of biomedical research [32]. The survey also asked “In some research animals are used instead of people. What do you believe could be a relevant reason to expose animals to research that we ourselves would not take part in?” Only a minority chose response options of “humans have higher moral status”, “humans have higher intelligence”, “animals do not have a soul”, or “animals suffer less than humans do”; most chose either “there are no relevant differences” (69.1% of patients, and 36.3% of scientists), or “there are other relevant differences” (11.5% of patients, and 43.7% of scientists) [32]. In a UK survey of scientists promoting AR, lay public, and animal welfarists, the support for AR (on a Likert scale of 7) was 5.33 (1.46), 3.57 (1.70), and 1.48 (0.87) respectively. These differences were largely explained by the scientists’ higher perception of lack of alternatives and of humans as superior, and lower perception of animal sentience [33]. Neither of these surveys asked for the amount of detail, or explored response to counterarguments, as in our survey.

This study has several limitations. Response rates for pediatricians and nurses/RTs were 46% and 61%; thus we cannot rule out biased participation in the survey. Arguments/counterarguments presented needed to be short and concise, and this may have left out important details that would have influenced the understanding of and response to the text. The moderate sample size from one University limits the generalizability of our results. Nevertheless, this is the first survey we are aware of that asks any group not just to consider whether they support AR; rather, to consider the most common arguments in the literature in favor of and against AR. Strengths of this study include the rigorous survey development process, and the inclusion of the most common and accepted arguments in the literature. Future study should determine the generalizability of our results.

In conclusion, when presented common arguments to justify AR, most respondents accepted ‘benefits’ arguments, and only a minority found the ‘characteristics of NHAs’ and ‘human exceptionalism’ arguments convincing. Most found the arguments to justify AR significantly weakened by common counterarguments, including those who initially found the ‘benefits’ arguments convincing. Engagement with and serious discussion of the arguments on both sides of the AR debate, deliberate extensive investigation of alternative research methods, and examination of the return on investment from AR and alternative research methods are potential ways forward in the debate about the moral permissibility of AR. We consider this survey an initial step toward this goal.
